# Single-molecule analysis of intracellular insulin granule behavior and its application to analyzing cytoskeletal dependence and pathophysiological implications

**DOI:** 10.3389/fphys.2023.1287275

**Published:** 2023-12-06

**Authors:** Hiroyasu Hatakeyama, Tomomi Oshima, Shinichiro Ono, Yuichi Morimoto, Noriko Takahashi

**Affiliations:** ^1^ Department of Physiology, Kitasato University School of Medicine, Sagamihara, Kanagawa, Japan; ^2^ International Research Center for Neurointelligence (WPI-IRCN), The University of Tokyo Institute for Advanced Study (UTIAS), The University of Tokyo, Bunkyo-ku, Tokyo, Japan; ^3^ Laboratory of Structural Physiology, Center for Disease Biology and Integrative Medicine, Faculty of Medicine, The University of Tokyo, Bunkyo-ku, Tokyo, Japan

**Keywords:** insulin granules, single-molecule analysis, cytoskeleton, actin, microtubules

## Abstract

**Introduction:** Mobilization of intracellular insulin granules to the plasma membrane plays a crucial role in regulating insulin secretion. However, the regulatory mechanisms of this mobilization process have been poorly understood due to technical limitations. In this study, we propose a convenient approach for assessing intracellular insulin granule behavior based on single-molecule analysis of insulin granule membrane proteins labeled with Quantum dot fluorescent nanocrystals.

**Methods:** This approach allows us to analyze intracellular insulin granule movement with subpixel accuracy at 33 fps. We tracked two insulin granule membrane proteins, phogrin and zinc transporter 8, fused to HaloTag in rat insulinoma INS-1 cells and, by evaluating the tracks with mean-square displacement, demonstrated the characteristic behavior of insulin granules.

**Results and discussion:** Pharmacological perturbations of microtubules and F-actin affected insulin granule behavior on distinct modalities. Specifically, microtubule dynamics and F-actin positively and negatively regulate insulin granule behavior, respectively, presumably by modulating each different behavioral mode. Furthermore, we observed impaired insulin granule behavior and cytoskeletal architecture under chronic treatment of high concentrations of glucose and palmitate. Our approach provides detailed information regarding intracellular insulin granule mobilization and its pathophysiological implications. This study sheds new light on the regulatory mechanisms of intracellular insulin granule mobilization and has important implications for understanding the pathogenesis of diabetes.

## 1 Introduction

Insulin secretion from pancreatic β-cells plays an important role in glucose homeostasis, and dysregulation of this process is directly involved in the etiology of diabetes (Kahn, 2001; Seino et al., 2011). In β-cells, insulin is stored in secretory granules during biogenesis. These granules undergo mobilization to and fuse with the plasma membrane in response to stimuli, such as high glucose, followed by exocytosis to the extracellular space (Orci et al., 1973; Rorsman and Rentröm, 2003). Our previous studies using two-photon excitation imaging of insulin granule exocytotic events in β-cells with extracellular polar-tracers demonstrated the occurrence of full flattening with the plasma membrane (full fusion exocytosis) with minor contributions from kiss-and-run exocytosis (∼6%) and sequential exocytosis (∼10%) (Takahashi et al., 2004; Hatakeyama et al., 2006), suggesting an important role for the mobilization of secretory granules in regulating insulin secretion. Therefore, the precise examination of insulin granule mobilization is critical for understanding the regulatory mechanisms involved in insulin secretion.

Several live-cell imaging techniques have been used to evaluate mobilization processes prior to exocytosis, such as the dynamic movement of insulin secretory granules (Pouli et al., 1998; Ivarsson et al., 2004; Tabei et al., 2013; Heaslip et al., 2014; Hoboth et al., 2015; Ferri et al., 2019; 2021). Specifically, total internal reflection fluorescence (TIRF) microscopy has made a major contribution to unveiling such dynamics including the dependence of cytoskeletal elements on the granule movement (Heaslip et al., 2014). This approach can detect the movement of individual insulin granules with a high signal-to-noise ratio and at high temporal resolution; however, it can only capture events that occur just beneath the plasma membrane, within approximately 100 nm inside the cell–coverslip interface. However, a significant portion of insulin granules also reside deeper inside cells beyond the TIRF zone (several micrometers from the plasma membrane) (Müller et al., 2020), insulin granule dynamics within such regions is thought to be also an important process for regulating insulin secretion. Indeed, insulin granule movement inside cells has also been evaluated using confocal microscopy combined with single-particle tracking techniques (Ivarsson et al., 2004; Tabei et al., 2013); however, such analyses employing conventional fluorescent molecules have limited temporal resolution. Furthermore, it is difficult to precisely track individual secretory granule movements with these approaches because the dense fluorescent signals resulting from the visualization of all fluorescent molecules makes it difficult to resolve individual granules. Recently, an approach based on image correlation spectroscopy for analyzing the dynamics of insulin granules without tracking individual granules has been demonstrated (Ferri et al., 2019; 2021). This approach is very useful for understanding the ensemble behavior of insulin granules in cells because it can extract structural and dynamic properties directly from images without single-particle techniques. Therefore, single-molecule analysis of insulin granules beyond the TIRF zone will advance a comprehensive understanding of insulin granule dynamics by combining of these techniques. We previously developed a simple, convenient, and nontoxic method for labeling intracellular molecules with fluorescent semiconductor nanoparticle Quantum dots (QDs) using HaloTag technology and electroporation and applied this method to the analysis of the intracellular behavior of myosin proteins (Hatakeyama et al., 2017). QDs are a suitable tool for single-particle tracking because of their extremely bright, single molecule fluorescence and high photostability. Our approach enables an expansion of the repertoire of proteins for which intracellular dynamics can be analyzed for single molecules, and insulin granules are an ideal example. Therefore, in the present study, we analyzed intracellular behavior of insulin granules by labeling insulin granule membrane proteins with QDs in INS-1 cells that exhibit glucose-responsive insulin secretion (Asfari et al., 1992) to analyze the role of cytoskeletal elements and their pathophysiological relevance.

## 2 Results

### 2.1 Labeling and tracking of intracellular insulin granules with QDs

**FIGURE 1:**
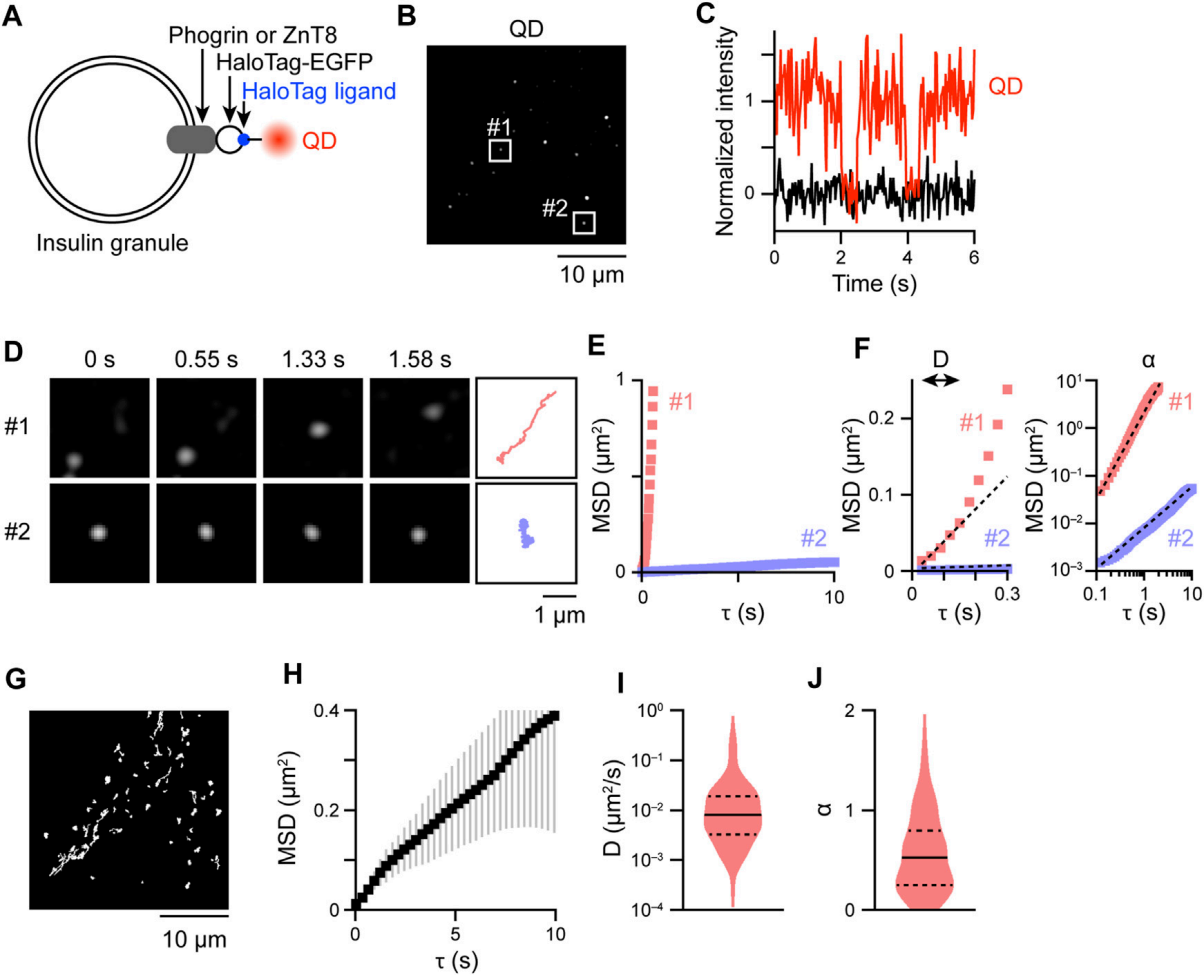
QD-labeling and tracking of insulin granules. **(A)** Schema for QD-labeling of insulin granule membrane proteins. **(B)** Snapshot of QD fluorescence in an INS-1 cell expressing HaloTag-ZnT8. The Laplacian of the Gaussian-filtered image. **(C)** Example of a QD fluorescence blinking event (red). The intensity in adjacent regions without QDs is also shown as background (black). **(D)** Changes in the position of QD signals in the boxed regions of **(B)**; fast- (#1) and slow-moving (#2) signals were selected. Trajectories of the particles are also shown at the far right. **(E)** MSD curves derived from the trajectories in **(D)**. **(F)** MSD curves with portions enlarged (τ ≤ 0.3 s) (left) or plotted on a log–log axis (right). Dashed lines represent the linear regression with Eq. 2 (left) and Eq. 3 (right). Based on the regression curves, D and α were obtained as: #1; D = 2.6 × 10^−2^ μm^2^/s, α = 1.7. #2; D = 1.3 × 10^−3^ μm^2^/s, α = 0.6. **(G)** Trajectories of all tracked particles (*n* = 140 particles) in a ce*ll* shown in **(B)**. **(H)** Mean MSD curve derived from the trajectories in **(G)**. Error bars are SEM. **(I,J)** Violin plots of log *D* and *α* derived from the trajectories in **(G)**. Solid and dashed lines represent the median and quartiles (upper and lower), respectively. The bathing solution for imaging experiments contained 2.8 mM D-glucose.

To track intracellular insulin granules with QDs, we selected to target the insulin granule membrane proteins, phogrin (Wasmeier and Hutton, 1996) and ZnT8 (Chimienti et al., 2004), as labeling molecules. Direct labeling of insulin itself with QDs would be very difficult, because it is necessary to penetrate the insulin granule membrane. Therefore, we incorporated HaloTag-enhanced green fluorescent protein (EGFP) into these membrane proteins at their cytoplasmic tail and expressed the fusion proteins in rat insulinoma INS-1 cells (Figure 1A). By staining these fusion proteins with HaloTag TMR ligand, localization of the proteins in mildly expressing cells was observed on punctate granule-like structures within the cytoplasm of the cells, which was completely different from that of control HaloTag that display diffuse signals throughout the cells (Supplementary Figure S1A). Such granule-like localization is quite similar to that of the insulin-HaloTag, and their diameters as measured by full width at half maximum were also similar among the proteins (Supplementary Figure S1B), the values of which were comparable to those obtained by previous electron microscopic investigations of insulin granules (Dean, 1973; Olofsson et al., 2002; Müller et al., 2020). Furthermore, many of such granule-like structures colocalized with insulin (Supplementary Figure S1C), showing that insulin granules can be tracked by tracking of these HaloTag proteins. Some structures apparently contain only one signal due to faint fluorescent signals (Supplementary Figure S1C, upper right). However, since we cannot completely rule out the possibility that some of the signals may represent non-colocalized phogrin/ZnT8 with insulin granules, we basically tracked the behavior of the two proteins for all types of experiments. No significant differences in behavior among insulin-HaloTag, phogrin-HaloTag, or ZnT8-HaloTag were observed (Supplementary Figure S1D). By electroporating QD-conjugated HaloTag ligands into the cells, we observed sparse bright spots of QD fluorescence (Figure 1B) as in our previous study (Hatakeyama et al., 2017). Blinking of most of the QD signals occurred in single steps (Figure 1C), indicating that most of the observed signals were derived from just one QD. QD fluorescence was collected for 30 s at 33 frames/s in EGFP-positive cells and analyzed the movement with sub-pixel accuracy (Figure 1D, Supplementary Movie S1). Movements varied from signal-to-signal, with some being mobile (Figure 1D, *upper*), whereas others stayed essentially in one position (Figure 1D, *lower*). To quantitatively analyze these movements, we calculated mean-square displacement (MSD) values for individual signals (Figure 1E), followed by evaluation with two scaling factors that were estimated from the MSD values, i.e., diffusion coefficient D that is estimated using the first 5 points (150 ms) of the MSD values and the anomalous exponent α that is estimated by the entire MSD values (Figure 1F, see *Materials and methods*) (Nelson et al., 2009; Pierobon et al., 2009; Hatakeyama et al., 2017). Diffusion coefficient D provides the dynamics of the motion for the brief time period that might be mainly regulated by diffusion (Hatakeyama et al., 2022), and the anomalous exponent α provides the characteristic of the motion for the entire trajectory: α = 0 for an immobile particle, α = 1 for a randomly diffusing particle, and α = 2 for a particle showing directional movement (Nelson et al., 2009). For analyzing intracellular behavior of insulin granules, we tracked all QD signals that could be tracked for more than 30 frames (Figure 1G) and compared the MSD curves and the distributions of D and α (Figures 1H–J) among the various treatment conditions. The D and α values were markedly varied among QD signals (Figures 1I, J), indicating heterogeneous behavior of insulin granules inside the cells. Note that the present analysis cannot extract the heterogeneity within individual trajectories. Methods such as segmentation with a short sliding time window can effectively capture such heterogeneity (Zajac et al., 2013), and further methods that can extract biologically relevant parameters without arbitrary thresholding would be necessary for more detailed analysis (see *Discussion*).

**FIGURE 2:**
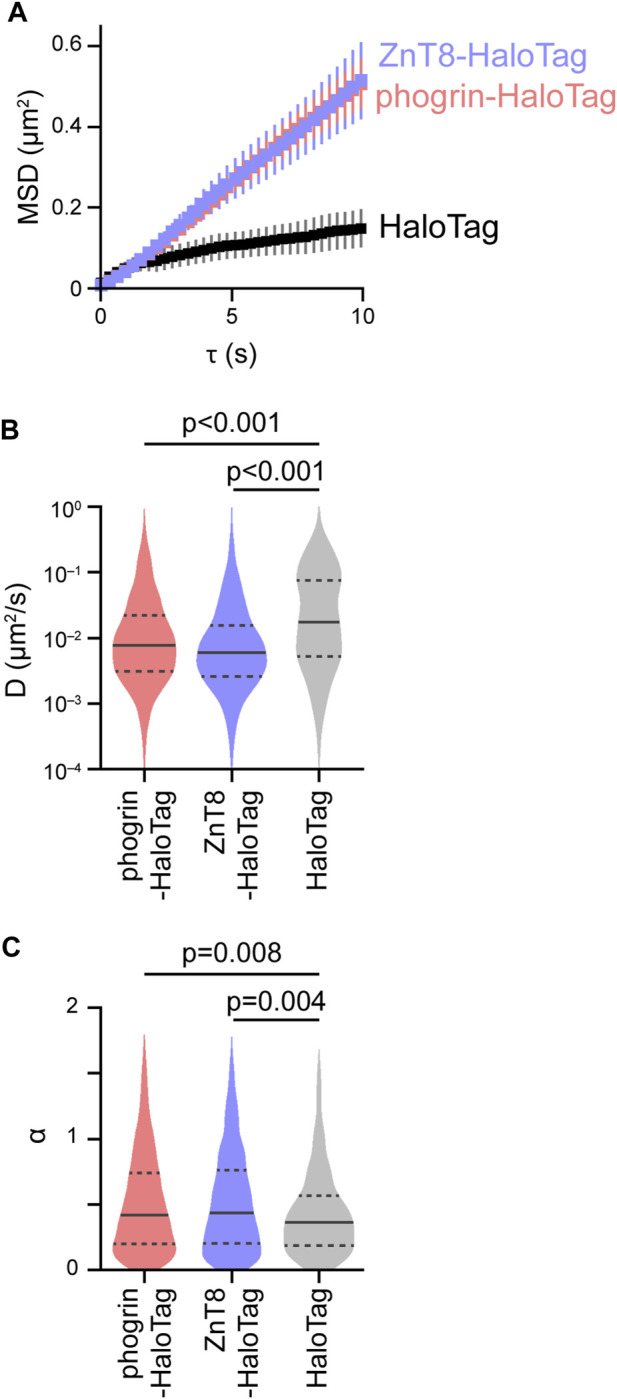
Evaluation of the intracellular movement of insulin granules. **(A)** Mean MSD curves in cells expressing control HaloTag (black), phogrin-HaloTag (red), and ZnT8-HaloTag (blue). Error bars represent SEM (*n* = 4-5 cells, 428–917 particles). **(B,C)** Violin plots of log *D* and *α*. Statistical significance was assessed as described in the Methods section.

The behavior of phogrin-HaloTag and ZnT8-HaloTag was almost the same (Figure 2A). Unexpectedly, the mobility of the control HaloTag was obviously smaller than that of phogrin-HaloTag and ZnT8-HaloTag despite smaller molecular weight of the control HaloTag (Figure 2A). Detailed analysis with D and α revealed characteristic differences in the movements of phogrin-HaloTag and ZnT8-HaloTag compared with that of control HaloTag, i.e., the fraction of molecules having a smaller D and a larger α was higher than that of control HaloTag (Figures 2B, C).

### 2.2 Cytoskeletal dependence of insulin granule movements

**FIGURE 3:**
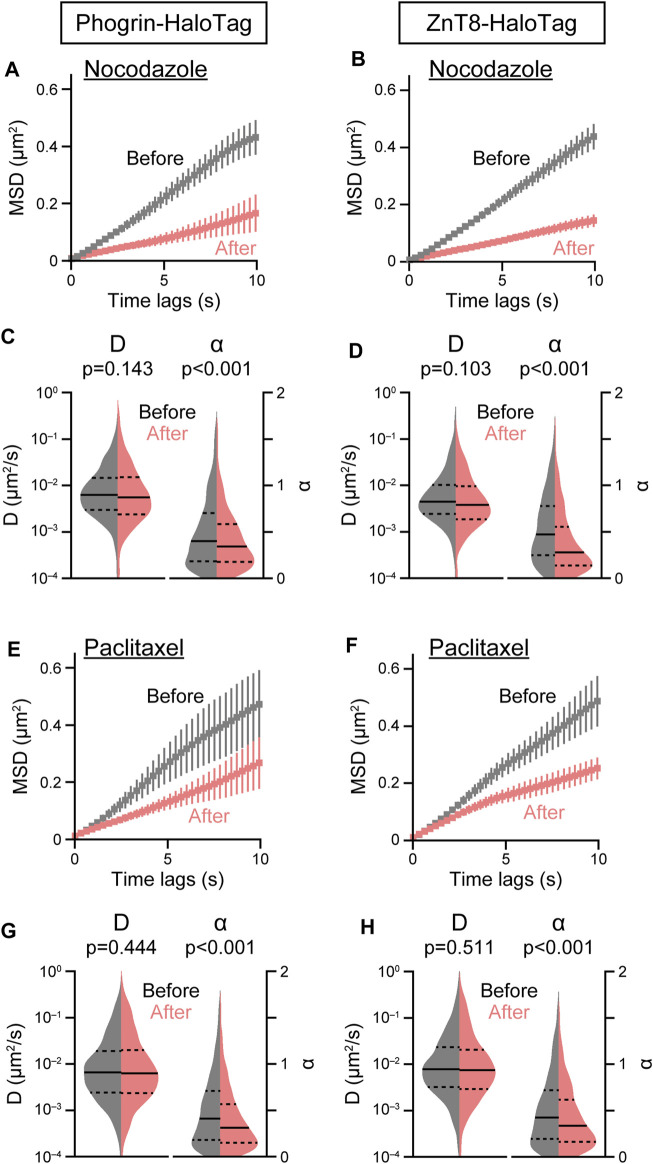
Effect of microtubule inhibitors on insulin granule movement. Effect of nocodazole (3 μM, **(A–D)** or paclitaxel (5 μM, **(E–H)** on mean MSD curves **(A,B,E,F)** and violin plots of *D* and **α** **(C,D,G,H)** in cells expressing phogrin-HaloTag (left) or ZnT8-HaloTag (right) before (gray) and 10 min after (red) treatment. Data with error bars are the mean ± SEM (*n* = 5–7 cells, 565–1,368 particles). Statistical significance was assessed as described in the Methods section.

**FIGURE 4:**
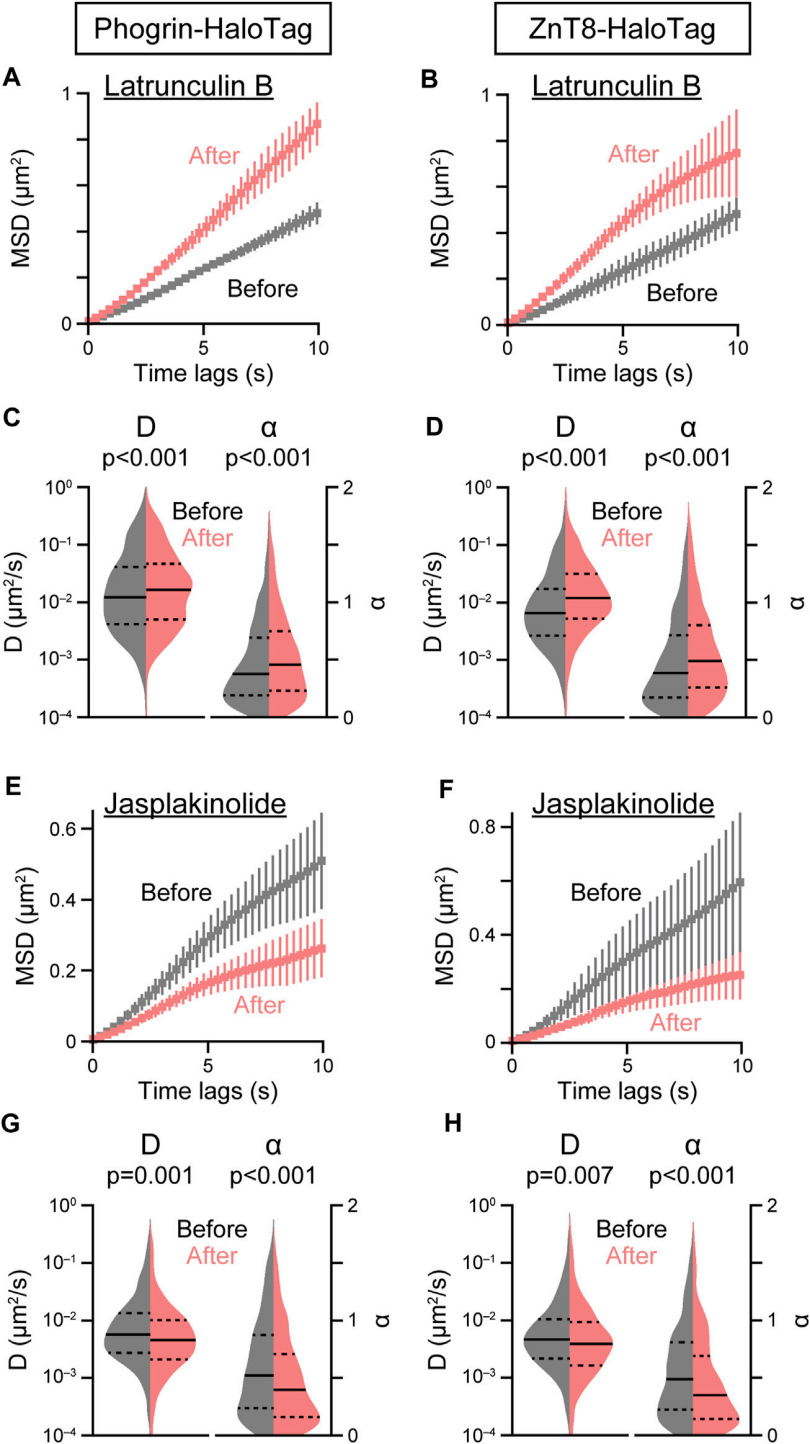
Effect of F-actin inhibitors on insulin granule movement. Effect of latrunculin B (10 μM, **(A–D)** or jasplakinolide (1 μM, **(E–H)** on the mean MSD curves **(A,B,E,F)** and violin plots of *D* and *α* **(C,D,G,H)** in cells expressing phogrin-HaloTag (left) or ZnT8-HaloTag (right) before (gray) and 5 min after (red) treatment. Data with error bars are the mean ± SEM (*n* = 4–9 cells, 539–1,554 particles). Statistical significance was assessed as described in the Methods section.

Cytoskeletal elements such as microtubules and F-actin play an important role in regulating trafficking and exocytosis of secretory granules containing peptide hormones including insulin. As an application of our single-particle analysis of insulin granule behavior, we investigated the role of cytoskeletal elements on the intracellular behavior of insulin granules by acute disturbance of microtubules and F-actin with pharmacological inhibitors. Destabilization of microtubules with nocodazole resulted in a marked suppression of both phogrin-HaloTag and ZnT8-HaloTag movements (Figures 3A, B). Nocodazole treatment significantly decreased α , but not D (Figures 3C, D). Interestingly, microtubule stabilization with paclitaxel also markedly suppressed both phogrin-HaloTag and ZnT8-HaloTag movements (Figures 3E, F), and the suppression was again mediated by a selective reduction in α (Figures 3G, H). These observations that two inhibitors with opposite actions similarly suppressed α suggest that microtubule dynamics as well as static microtubule architecture play a role in regulating insulin granule behavior. Regarding F-actin, we found destabilization of F-actin with latrunculin B resulted in a marked acceleration in both phogrin-HaloTag and ZnT8-HaloTag movements (Figures 4A, B). In contrast to microtubule inhibition, latrunculin B treatment significantly increased both D and α (Figures 4C, D). In contrast, stabilization of F-actin with jasplakinolide suppressed movement (Figures 4E, F), which was mediated by a significant reduction in both D and α (Figures 4G, H), suggesting that F-actin plays an inhibitory role on insulin granule movement. We also confirmed these observations by tracking the insulin-HaloTag itself in cells and staining the insulin-HaloTag with HaloTag TMR ligand, although the time resolution was limited (Supplementary Figure S2).

### 2.3 Pathophysiological relevance of cytoskeleton-dependent insulin granule behavior

**FIGURE 5:**
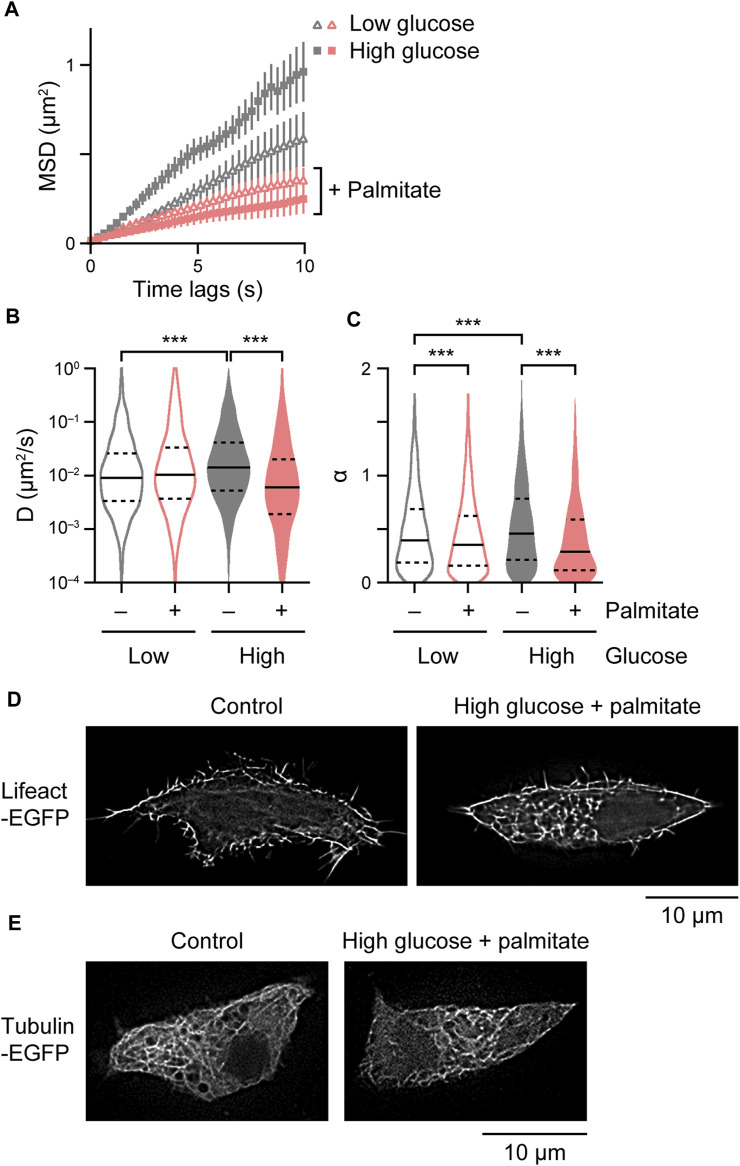
Derangement of insulin granule behavior and cytoskeletal architectures under glucolipotoxic models. **(A–C)** Mean MSD curves **(A)** and violin plots of *D* **(B)** and *α* **(C)** in cells expressing ZnT8-HaloTag treated with 2.8 mM or 33.3 mM D-glucose and/or 0.4 mM palmitate for 16–20 h (*n* = 18–28 cells, 2,363–3,160 particles). Statistical significance was assessed as described in the Materials and methods section. ***p < 0.001. **(D,E)** SRRF images in cells expressing Lifeact-EGFP **(D)** and tubulin-EGFP **(E)** treated with 2.8 mM (left) or 33.3 mM D-glucose and 0.4 mM palmitate for 16–20 h (right).

We finally analyzed intracellular behavior of insulin granules under glucolipotoxic conditions by chronically treating INS-1 cells with high concentrations of glucose and palmitate (Pasquier et al., 2019). High glucose itself facilitated insulin granule behavior (Figures 5A–C). Palmitate suppressed insulin granule behavior regardless of glucose concentration, but more strongly in the presence of high concentration of glucose (Figures 5A–C). Interestingly, the cells chronically treated with high glucose and palmitate displayed massive F-actin architectures (Figure 5D), which may at least in part explain the suppression of insulin granule behavior. We observed no obvious changes in microtubule architectures in cells expressing tubulin-EGFP treated with high glucose and palmitate (Figure 5E).

## 3 Discussion

In the current study, we proposed a high precision approach for analyzing insulin granule behavior located in relatively deep subcellular regions based on single molecule imaging of QDs of the insulin granule membrane proteins, phogrin and ZnT8. This approach is based on our previously reported study, in which we analyzed intracellular behavior of myosin motors by taking advantage of the relatively simple methodology involving the electroporation of cells expressing HaloTag-fused proteins seeded on standard glass-bottom dishes (Hatakeyama et al., 2017). Although tracking of intracellular insulin granules in live cells employing conventional fluorescent molecules have been performed in this (Supplementary Figures S1, S2) and previous studies (Ivarsson et al., 2004; Tabei et al., 2013), temporal resolution of these approaches was inevitably limited compared with our proposed approach using QDs. This limitation has resulted in the preferential analysis of relatively slow-moving granules. Furthermore, images acquired with such conventional molecules tend to contain crowded signals derived from many fluorescent molecules within limited regions, making it difficult to precisely resolve individual granules. Methods based on TIRF microscopy (Heaslip et al., 2014; Hoboth et al., 2015) can analyze insulin granule behavior at high temporal resolution, but this approach also has limitations as it can only detect granule movement within the subplasmalemmal space. Our current approach overcomes these limitations and enables the analysis of insulin granule behavior deep inside cells with high temporal resolution. Although our current analysis is mainly based on ensemble behavior of intracellular insulin granules as in analysis based on image correlation spectroscopy (Ferri et al., 2019; 2021), our HaloTag/QD-based approach allows us to directly compare intracellular behavior between molecules localized to punctate structures, such as insulin granule membrane proteins, and molecules showing a diffuse localization, such as the control HaloTag, with the same accuracy without any assumptions (Figure 2; Supplementary Figure S1). In the present study, insulin granules exhibited a grossly facilitative behavior compared with the control HaloTag proteins, but with smaller D and larger α (Figure 2), suggesting the presence of unique regulatory mechanisms of insulin granule behavior. Unexpectedly restricted behavior of control HaloTag may be attributed to several possibilities, including 1) nonspecific binding of HaloTag-ligand QDs, and 2) our current acquisition conditions cannot track freely diffusing molecules sufficiently (and therefore preferentially track slow-diffusing molecules). For the former possibility, although it is difficult to calculate the fraction of nonspecifically bound QDs, we previously observed that the cells that had been allowed to incorporate HaloTag ligand-QDs can be further labeled with another HaloTag ligand (Hatakeyama et al., 2017). This observation suggests that the number of HaloTag proteins expressed in the cells are larger than that of HaloTag ligand-QDs incorporated into the cells, presumably lowering the possibility of nonspecific QD-binding. The latter possibility would be highly possible since the diffusion coefficient of molecules like EGFP in mammalian cells examined with FRAP and FCS is over 10 μm^2^/s (Elsner et al., 2003; Sprague et al., 2004), of which values cannot be captured with our current approach (Figure 2). The fact that α values of control HaloTag were <1 may further suggest this possibility. Faster image acquisition may make it possible to track such freely diffusing molecules more accurately. Nevertheless, it would be important that the behavioral characteristics of insulin granule-resident proteins detected were quite different from those of control HaloTag proteins. We also observed that less than 5% of insulin granules exhibited directed motion (insulin granules with α values of >1.5), whereas a previous study with TIRF microscopy demonstrated that >10% of the granules had α values >1.5 within the subplasmalemmal space (Heaslip et al., 2014). Therefore, distinct mechanisms regulating intracellular insulin granule behavior should exist between the intracellular space and on the plasma membrane, indicating the importance of analyzing their behavior beyond TIRF zones as well as on the plasma membrane. The involvement of cytoskeletal elements on insulin secretion mechanisms was first proposed over 50 years ago (Lacy et al., 1968), when extensive studies revealed the critical link of cytoskeletal elements with secretory processes in β-cells (Howell and Tyhurst, 1982). These studies demonstrated that microtubules serve as a guiding cytoskeleton from the granule synthesis site to the cell periphery, which is mediated by kinesin motors (Malaisse-Lagae et al., 1979; Varadi et al., 2003; 2005), whereas the periphery actin network regulates granule transport mediated by myosin motors (Thurmond et al., 2003; Ivarsson et al., 2005; Varadi et al., 2005). If the microtubules play a simple guiding role, destabilizing and stabilizing the structures will suppress or facilitate granule behavior, respectively. However, our current observations demonstrate that both destabilization with nocodazole and stabilization with paclitaxel acutely suppressed granule behavior. These results indicate a central role for microtubules undergoing cycles of growth and shortening as well as static structures on the behavior. Such inhibition was mediated by the selective suppression of α , suggesting that microtubules dynamics positively regulate the behavior that is possible to affect the entire trajectory. These observations are consistent with previous imaging analyses of the movement beneath the plasma membrane by TIRF microscopy, although with longer treatment with inhibitors (Heaslip et al., 2014). Therefore, microtubules may act as a “dynamic” guide to transport granules between the cell center and the periphery. Recent FIB-SEM analysis in beta-cells also demonstrated important active roles of microtubules in transporting insulin granules, since insulin granules were enriched close to microtubules independently of glucose concentration, with ∼1/3 of the granules suggesting direct interaction with microtubules (Müller et al., 2020). Regarding F-actin, we demonstrated that destabilization with latrunculin and stabilization with jasplakinolide facilitated and suppressed granule behavior, respectively, indicating that F-actin acts as a physical barrier to granule behavior. Although a previous study proposed that cortical actin acts as a barrier (Aunis and Bader, 1988), our findings indicate that “intracellular” actin also function as a physical barrier. Our observation that actin inhibitors affect both D and α also supports this hypothesis. We actually observed obvious intracellular actin dynamics as well as on the cell surface and some interrelationships between intracellular actin dynamics and insulin granule behavior (Hatakeyama et al. unpublished data). Detailed mechanisms involved in these cytoskeletal dependencies remain unclear, and further analyses will be necessary to clarify these relationships. Such cytoskeletal dependance of insulin granule behavior is possible to be involved in certain cases of pathophysiology including glucolipotoxicity, since deranged intracellular granule behavior and F-actin architectures under glucolipotoxic conditions were clearly observed. Although molecular mechanisms involving such F-actin derangement are unclear, previous study demonstrated that palmitate can induce changes in actin dynamics that are dependent on mitochondrial oxidative stress or phospholipase C signaling (Xu et al., 2015). Further analyses will progress understanding the pathogenesis of glucolipotoxic conditions.

We should note that there is room for improvement to our current approach. In the present study, we analyzed insulin granule movement in an INS-1 insulinoma cell line, but physiologically, it would be critical to analyze higher-order samples, such as pancreatic islet cells. We have successfully introduced QDs into pancreatic islet cells (Hatakeyama et al. unpublished data). In terms of analysis in these higher-order cells, three-dimensional analyses of granule movement would be desirable, whereas our current approach acquires time series data in two-dimensional images. Although three-dimensional image acquisition with high temporal resolution is challenging, various methods for three-dimensional tracking have been proposed (Shen et al., 2017; Gardini et al., 2018; Liebel et al., 2020). Combining these techniques with our approach will provide further insight into the behavior of insulin granules. Physiologically, important questions remain including 1) how physiological stimuli, such as glucose and incretin hormones, affect insulin granule behavior and 2) how granule behavior and exocytosis are related. For the former issue, prolonged treatment of high glucose facilitated insulin granule behavior (although it should be noted that the concentration was supraphysiological) (Figure 5), indicating glucose can regulate insulin granule behavior. Insulin granule behavior just beneath the plasma membrane has been shown to be facilitated by acute glucose stimulation (Heaslip et al., 2014; Hoboth et al., 2015), and it is imperative to use our approach to analyze the effects of acute glucose stimulation on insulin granule behavior and cytoskeletal dynamics inside the cells. For the latter issue, because QDs that have a broad absorption spectrum and a large Stokes shift can be visualized concurrently with various other fluorescent molecules (Hatakeyama et al., 2017; 2022), simultaneous detection of intracellular granule movement and exocytotic processes may be possible by employing QDs and extracellular polar tracers (Takahashi et al., 2004; Hatakeyama et al., 2006; Shin et al., 2018), respectively. Finally, although our estimation of the behavioral parameters, D and α , in the present study were based on the assumption that each single trajectory contains only one mode of behavior, in the real situation, it is highly possible that a single trajectory contains multiple modes of behavior. Combining methods for the unbiased detection of multiple modes, including those employing artificial intelligence (Das et al., 2009; Pinaud et al., 2009; Persson et al., 2013; Monnier et al., 2015; Matsuda et al., 2018; Newby et al., 2018; Arts et al., 2019) with our approach, may provide a solution for precisely analyzing such behavior. It has been suggested that stable docking of secretory granules to the plasma membrane is not an essential factor for insulin granule exocytosis (Gomi et al., 2005; Hatakeyama et al., 2007; Kasai et al., 2008; Takahashi et al., 2015). Thus, further insight into the dynamic processes of insulin granule mobilization will provide a better understanding of the regulatory mechanisms of insulin secretion.

Overall, our approach can provide detailed information regarding intracellular insulin granule mobilization, and we believe our approach will shed new light on the regulatory mechanisms of intracellular insulin granule mobilization and has important implications for understanding the pathogenesis of diabetes.

## 4 Materials and methods

### 4.1 Cell lines

INS-1 cells were grown at 37°C in a 5% CO_2_/95% air in RPMI1640 containing 10% FBS, 1 mM sodium pyruvate, 50 μM 2-mercaptoethanol, 10 mM HEPES, and 1% penicillin/streptomycin (100 U/mL penicillin and 100 μg/mL streptomycin). Plasmids were transfected into cells via reverse transfection method with Lipofectamine 3,000 and Opti-MEM I in glass-bottom dishes (thickness 0.17 mm, Matsunami-glass). In some experiments, the cells were stained with 0.5 μM HaloTag TMR ligand (Promega). All imaging experiments were performed 24 h after transfection.

### 4.2 Plasmids

Human insulin transcript variant 1 (GenBank accession #AB587580) was amplified from the pFN21AB8864 vector (Promega) with primers 5′-CGA​TGA​ATT​CGC​CAC​CAT​GGC​CCT​GTG​GAT​GCG​C-3' (forward) and 5′-TCG​TCT​CGA​GGT​TGC​AGT​AGT​TCT​CCA​GC-3' (reverse). Rat phogrin (GenBank accession #z50735) was amplified from phogrin-EGFP-N1 (Pouli et al., 1998) (kindly provided by Prof. Guy A. Rutter) with primers 5′-CTT​CGA​ATT​CGC​CAC​CAT​GGG​GCT​ACC​GCT​CCC-3' (forward) and 5-CGA​CCT​CGA​GCT​GGG​GAA​GGG​CCT​TCA​G-3' (reverse). The human zinc transporter transcript variant 1 (GenBank accession #NM173851) was amplified from the Zinc transporter 8 human tagged ORF clone (Origene) with primers 5′-GCC​GAA​TTC​GCC​ACC​ATG​GAG​TTT​CTT​GAA​AGA​ACG-3' (forward) and 5′-GCG​TCT​CGA​GGT​CAC​AGG​GGT​CTT​CAC​AG-3' (reverse). All three constructs were modified with an EcoRI site and a Kozak sequence at the N-terminus and an XhoI site at the C-terminus. The amplified products were digested with EcoRI/XhoI and inserted into the multiple cloning site of the pHTC HaloTAG CMV neo vector to generate the pHTC-insulin-HaloTag, pHTC-phogrin-HaloTag, and pHTC-ZnT8-HaloTag vectors. EGFP was amplified from the phogrin-EGFP-N1 vector as a template with primers 5′-GGC​TGT​CTA​CTC​TGG​AGA​TTT​CCG​GTG​GCG​GCA​GCG​GAG​GAT​CCG​GCG​GAA​CAT​GTA​CAC​CGC​TGA​GCG​CCA​CCA​TGG​TGA​GCA​AGG​GCG​AG-3' (forward) and 5′-AGT​CGC​GGC​CGC​TTT​ACT​TGT​AC-3' (reverse), and an AccI site and linker were added. The amplified product was digested with AccI/NotI and inserted into the C-terminus of the pHTC-phogrin-HaloTag vector to create the pHTC-phogrin-HaloTag-EGFP vector. Then, the pHTC-phogrin-HaloTag-EGFP vector was digested with EcoRI/XhoI, and phogrin was replaced with ZnT8 to generate the pHTC-ZnT8-HaloTag-EGFP vector.

### 4.3 Conjugation of the HaloTag ligand with QDs and electroporation

Conjugation of the HaloTag ligand with QDs was performed with a HaloTag succinimidyl ester (O2) ligand and Qdot ITK amino (PEG) QD655 as previously described (Hatakeyama et al., 2017) with slight modification. For electroporation of the QD-conjugated HaloTag ligand, the cells on the glass-bottom dish were immersed in Opti-MEM I containing 5 nM of QD-conjugated HaloTag ligand and electroporated with a CUY21EDIT II electroporator and an LF513-5 electrode by applying a pulse of 200 V for 10 ms, followed by five pulses at ±30 mV for 10 ms at 50 ms intervals. The cells were then washed and cultured overnight in culture medium.

### 4.4 Immunofluorescence and colocalization analysis

Immunofluorescence was performed with standard protocols. The cells labelled with HaloTag TMR ligand were fixed, permeabilized, treated with anti-insulin antibodies (Cell Signaling), and stained with Alexa 647-conjugated anti-rabbit IgG (Abcam). For colocalization analysis, background fluorescence of the acquired images was measured within the cell-free area adjacent to the cell of interest and its mean + 3SD value was subtracted from the entire image. Colocalization coefficient was obtained with Coloc2 plug-in of Fiji ImageJ (Schindelin et al., 2012).

### 4.5 Imaging and particle tracking

All imaging experiments were performed with an inverted microscope (IX81 or IX83) equipped with an EMCCD camera (iXon ultra), a spinning disk confocal unit (CSU-X1), a z-drift compensator (IX81-ZDC2 or IX3-ZDC2), SRRF-stream module (Andor) and an oil-immersion objective lens (UPLSAPO100xO, NA1.4) at approximately 30°C with both a stage heater and a lens heater (TOKAI HIT). Images were acquired with iQ software (Andor). The bathing solution for imaging experiments consisted of 150 mM NaCl, 5 mM KCl, 2 mM CaCl_2_, 1 mM MgCl_2_, 10 mM HEPES-NaOH (pH 7.4) and 2.8 mM D-glucose unless otherwise indicated. For QD imaging, excitation was 532 or 555 nm, and the fluorescence was acquired through a 655/15 bandpass filter (Semrock) at 33 frames/s for 30 s. Pixel sizes were approximately 0.082 μm/pixel (Hatakeyama et al., 2023). For TMR imaging, excitation was 532 or 555 nm, and the fluorescence was acquired through a 594/40 bandpass filter (Semrock) at 2 frames/s for 120 s (Ono et al., 2023). All imaging was performed at a depth of 1-2 μm above the glass surface. Single-particle tracking was performed with the TrackMate plugin (Tinevez et al., 2017) of Fiji ImageJ with subpixel accuracy. For QDs, we used LoG detector with blob diameter of 12 pixels, simple LAP tracker with linking, gap-closing max distances of 6 pixels, gap-closing max frame of 5, selected spots with Quality values > 2, and tracks containing >30 positions for subsequent analysis. For TMR, we used LoG detector with blob diameter of 6 pixels, simple LAP tracker with linking max distances of 6 pixels, set Quality values in which there were no spots with Signal-to-Noise Ratio values of <0, and selected tracks containing >10 positions for subsequent analysis. Based on the tracks, we calculated the MSD of the particles with a slightly modified script of the @msdanalyzer (Tarantino et al., 2014). MSD was calculated as follows:

$$MSD\left(\tau \right)=\frac{1}{N-\frac{\tau }{\Delta t}}\sum_{i=1}^{N-\frac{\tau }{\Delta t}}{\left|p_{i+\frac{\tau }{\Delta t}}+p_{i}\right|}^{2}$$ (1)

where τ represents all accessible time lags, N is the total number of frames in the measured trajectories, Δt is the time interval of successive images, and $p_{i}$ is the coordinate of the molecule in the time frame i . Because $MSD\left(\tau \right)$ values are known to be proportional to 2nDτ when τ is small, where n and D are the dimension and the diffusion coefficient, respectively, we estimated the diffusion coefficient of individual trajectories obtained from two-dimensional images by fitting the first five points of the corresponding $MSD\left(\tau \right)$ curve to the formula:

$$MSD\left(\tau \right)=4D\tau +C$$ (2)

where C is the position error. $MSD\left(\tau \right)$ also typically exhibits approximate power-law behavior as follows:

$$MSD\left(\tau \right)\propto \tau^{\alpha }$$ (3)

The exponent α , which can be obtained as a slope of the $MSD\left(\tau \right)$ values plotted on a log–log axis, provides the characteristics of the motion. For superresolution radial fluctuation (SRRF) imaging (Gustafsson et al., 2016) of EGFP, excitation was at 470 or 488 nm, and the fluorescence was acquired through 525/50 bandpass filter (Semrock) at 20 frames/s for 3 s. SRRF reconstruction was performed employing NanoJ-SRRF plugin of Fiji ImageJ. The reconstruction parameters were: ring radium 0.25, radiality magnification 5, axes in ring 6, frames per time-point 60, and TRA with gradient smoothing and intensity weighting.

### 4.6 Preparation of palmitate/BSA-complex solution

Palmitate/BSA-complex solution was prepared as described previously (Cousin et al., 2001). Briefly, 100 mM palmitate was prepared in 0.1 M NaOH at 70°C. In parallel, 5% FFA-free BSA solution was prepared in serum-free RPMI1640 medium at 56°C. One mM palmitate/5% BSA solution was prepared by adding an appropriate amount of palmitate solution dropwise to 5% BSA solution at 56°C, then vortex mixed for 10 s followed by a further 10-min incubation at 56°C. The palmitate/BSA complex solution was filtered and diluted 1:5 in serum-free RPMI1640 to a final concentration of 0.4 mM palmitate/1% BSA. Imaging experiments were performed after 16–20 h treatment of palmitate/BSA in the continuous presence of palmitate/BSA.

### 4.7 Statistical analysis

The statistical significance of the observed differences between two conditions was determined by the randomization test using Shiny apps (https://huygens.science.uva.nl/) at https://thenode.biologists.com/user-friendly-p-values/research/. A *p*-value less than 0.05 was considered statistically significant.

## Data Availability

The raw data supporting the conclusion of this article will be made available by the authors, without undue reservation.

## References

[B1] Arts M. Smal I. Paul M. W. Wyman C. Meijering E. (2019). Particle mobility analysis using deep learning and the moment scaling spectrum. Sci. Rep. 9, 17160. 10.1038/s41598-019-53663-8 31748591 PMC6868130

[B2] Asfari M. Janjic D. Meda P. Li G. Halban P. A. Wollheim C. B. (1992). Establishment of 2-mercaptoethanol-dependent differentiated insulin-secreting cell lines. Endocrinology 130, 167–178. 10.1210/endo.130.1.1370150 1370150

[B3] Aunis D. Bader M. F. (1988). The cytoskeleton as a barrier to exocytosis in secretory cells. J. Exp. Biol. 139, 253–266. 10.1242/jeb.139.1.253 3062121

[B4] Chimienti F. Devergnas S. Favier A. Seve M. (2004). Identification and cloning of a beta-cell-specific zinc transporter, ZnT-8, localized into insulin secretory granules. Diabetes 53, 2330–2337. 10.2337/diabetes.53.9.2330 15331542

[B5] Cousin S. P. Hugl S. R. Wrede C. E. Kajio H. Myers M. G. Rhodes C. J. (2001). Free fatty acid-induced inhibition of glucose and insulin-like growth factor I-induced deoxyribonucleic acid synthesis in the pancreatic beta-cell line INS-1. Endocrinology 142, 229–240. 10.1210/endo.142.1.7863 11145586

[B6] Das R. Cairo C. W. Coombs D. (2009). A hidden Markov model for single particle tracks quantifies dynamic interactions between LFA-1 and the actin cytoskeleton. PLoS Comput. Biol. 5, e1000556. 10.1371/journal.pcbi.1000556 19893741 PMC2768823

[B7] Dean P. M. (1973). Ultrastructural morphometry of the pancreatic β-cell. Diabetologia 9, 115–119. 10.1007/BF01230690 4577291

[B8] Elsner M. Hashimoto H. Simpson J. C. Cassel D. Nilsson T. Weiss M. (2003). Spatiotemporal dynamics of the COPI vesicle machinery. EMBO Rep. 4, 1000–1004. 10.1038/sj.embor.embor942 14502225 PMC1326400

[B9] Ferri G. Digiacomo L. Lavagnino Z. Occhipinti M. Bugliani M. Cappello V. (2019). Insulin secretory granules labelled with phogrin-fluorescent proteins show alterations in size, mobility and responsiveness to glucose stimulation in living beta-cells. Sci. Rep. 9, 2890. 10.1038/s41598-019-39329-5 30814595 PMC6393586

[B10] Ferri G. Tesi M. Pesce L. Bugliani M. Grano F. Occhipinti M. (2021). Spatiotemporal correlation spectroscopy reveals a protective effect of peptide-based GLP-1 receptor agonism against lipotoxicity on insulin granule dynamics in primary human beta-cells. Pharmaceutics 13, 1403. 10.3390/pharmaceutics13091403 34575477 PMC8464798

[B11] Gardini L. Calamai M. Hatakeyama H. Kanzaki M. Capitanio M. Pavone F. S. (2018). Three-dimensional tracking of quantum dot-conjugated molecules in living cells. Methods Mol. Biol. 1814, 425–448. 10.1007/978-1-4939-8591-3_26 29956248

[B12] Gomi H. Mizutani S. Kasai K. Itohara S. Izumi T. (2005). Granuphilin molecularly docks insulin granules to the fusion machinery. J. Cell Biol. 171, 99–109. 10.1083/jcb.200505179 16216924 PMC2171228

[B13] Gustafsson N. Culley S. Ashdown G. Owen D. M. Pereira P. M. Henriques R. (2016). Fast live-cell conventional fluorophore nanoscopy with ImageJ through super-resolution radial fluctuations. Nat. Commun. 7, 12471. 10.1038/ncomms12471 27514992 PMC4990649

[B14] Hatakeyama H. Kishimoto T. Nemoto T. Kasai H. Takahashi N. (2006). Rapid glucose sensing by protein kinase A for insulin exocytosis in mouse pancreatic islets. J. Physiol.-Lond. 570, 271–282. 10.1113/jphysiol.2005.096560 16284079 PMC1464314

[B15] Hatakeyama H. Kobayashi K. Kanzaki M. (2022). Three live-imaging techniques for comprehensively understanding the initial trigger for insulin-responsive intracellular GLUT4 trafficking. iScience 25, 104164. 10.1016/j.isci.2022.104164 35434546 PMC9010770

[B57] Hatakeyama H. Ono S. Oshima T. Takahashi N. (2023). Role of F-actin in intracellular insulin granule behavior analyzed by single-particle tracking. Proceedings of the 100th Annual Meeting of The Physiological Society of Japan. J. Physiol. Sci. 73 (Suppl 1), 11. 10.1186/s12576-023-00867-3

[B16] Hatakeyama H. Nakahata Y. Yarimizu H. Kanzaki M. (2017). Live-cell single-molecule labeling and analysis of myosin motors with quantum dots. Mol. Biol. Cell 28, 173–181. 10.1091/mbc.E16-06-0413 28035048 PMC5221621

[B17] Hatakeyama H. Takahashi N. Kishimoto T. Nemoto T. Kasai H. (2007). Two cAMP-dependent pathways differentially regulate exocytosis of large dense-core and small vesicles in mouse beta-cells. J. Physiol. 582, 1087–1098. 10.1113/jphysiol.2007.135228 17510178 PMC2075257

[B18] Heaslip A. T. Nelson S. R. Lombardo A. T. Beck Previs S. Armstrong J. Warshaw D. M. (2014). Cytoskeletal dependence of insulin granule movement dynamics in INS-1 beta-cells in response to glucose. PLoS One 9, e109082. 10.1371/journal.pone.0109082 25310693 PMC4195697

[B19] Hoboth P. Muller A. Ivanova A. Mziaut H. Dehghany J. Sonmez A. (2015). Aged insulin granules display reduced microtubule-dependent mobility and are disposed within actin-positive multigranular bodies. Proc. Natl. Acad. Sci. U A 112, E667–E676. 10.1073/pnas.1409542112 PMC4343180 25646459

[B20] Howell S. L. Tyhurst M. (1982). Microtubules, microfilaments and insulin-secretion. Diabetologia 22, 301–308. 10.1007/BF00253571 6284576

[B21] Ivarsson R. Jing X. Waselle L. Regazzi R. Renström E. (2005). Myosin 5a controls insulin granule recruitment during late-phase secretion. Traffic 6, 1027–1035. 10.1111/j.1600-0854.2005.00342.x 16190983

[B22] Ivarsson R. Obermuller S. Rutter G. A. Galvanovskis J. Renström E. (2004). Temperature-sensitive random insulin granule diffusion is a prerequisite for recruiting granules for release. Traffic 5, 750–762. 10.1111/j.1600-0854.2004.00216.x 15355511

[B23] Kahn S. E. (2001). Clinical review 135: the importance of beta-cell failure in the development and progression of type 2 diabetes. J. Clin. Endocrinol. Metab. 86, 4047–4058. 10.1210/jcem.86.9.7713 11549624

[B24] Kasai K. Fujita T. Gomi H. Izumi T. (2008). Docking is not a prerequisite but a temporal constraint for fusion of secretory granules. Traffic 9, 1191–1203. 10.1111/j.1600-0854.2008.00744.x 18397364

[B25] Lacy P. E. Howell S. L. Young D. A. Fink C. J. (1968). New hypothesis of insulin secretion. Nature 219, 1177–1179. 10.1038/2191177a0 4877537

[B26] Liebel M. Ortega Arroyo J. Beltran V. S. Osmond J. Jo A. Lee H. (2020). 3D tracking of extracellular vesicles by holographic fluorescence imaging. Sci. Adv. 6, eabc2508. 10.1126/sciadv.abc2508 33148645 PMC7673696

[B27] Malaisse-Lagae F. Amherdt M. Ravazzola M. Sener A. Hutton J. C. Orci L. (1979). Role of microtubules in the synthesis, conversion, and release of (pro)insulin. A biochemical and radioautographic study in rat islets. J. Clin. Invest. 63, 1284–1296. 10.1172/JCI109423 376557 PMC372077

[B28] Matsuda Y. Hanasaki I. Iwao R. Yamaguchi H. Niimi T. (2018). Estimation of diffusive states from single-particle trajectory in heterogeneous medium using machine-learning methods. Phys. Chem. Chem. Phys. 20, 24099–24108. 10.1039/c8cp02566e 30204178

[B29] Monnier N. Barry Z. Park H. Y. Su K.-C. Katz Z. English B. P. (2015). Inferring transient particle transport dynamics in live cells. Nat. Methods 12, 838–840. 10.1038/nmeth.3483 26192083 PMC4733533

[B30] Müller A. Schmidt D. Xu C. S. Pang S. D’Costa J. V. Kretschmar S. (2020). 3D FIB-SEM reconstruction of microtubule–organelle interaction in whole primary mouse β cells. J. Cell Biol. 220, e202010039. 10.1083/jcb.202010039 PMC7748794 33326005

[B31] Nelson S. R. Ali M. Y. Trybus K. M. Warshaw D. M. (2009). Random walk of processive, quantum dot-labeled myosin Va molecules within the actin cortex of COS-7 cells. Biophys. J. 97, 509–518. 10.1016/j.bpj.2009.04.052 19619465 PMC2711322

[B32] Newby J. M. Schaefer A. M. Lee P. T. Forest M. G. Lai S. K. (2018). Convolutional neural networks automate detection for tracking of submicron-scale particles in 2D and 3D. Proc. Natl. Acad. Sci. U A 115, 9026–9031. 10.1073/pnas.1804420115 PMC6130393 30135100

[B33] Olofsson C. S. Gopel S. O. Barg S. Galvanovskis J. Ma X. Salehi A. (2002). Fast insulin secretion reflects exocytosis of docked granules in mouse pancreatic B-cells. Pflugers Arch. 444, 43–51. 10.1007/s00424-002-0781-5 11976915

[B58] Ono S. Hatakeyama H. Oshima T. Takahashi N. (2023). Live-imaging analysis of F-actin actions on intracellular insulin granule behavior. Proceedings of the 100th Annual Meeting of The Physiological Society of Japan. J. Physiol. Sci. 73 (Suppl 1), 11. 10.1186/s12576-023-00867-3

[B34] Orci L. Amherdt M. Malaisse-Lagae F. Rouiller C. Renold A. E. (1973). Insulin release by emiocytosis: demonstration with freeze-etching technique. Science 179, 82–84. 10.1126/science.179.4068.82 4565325

[B35] Pasquier A. Vivot K. Erbs E. Spiegelhalter C. Zhang Z. Aubert V. (2019). Lysosomal degradation of newly formed insulin granules contributes to beta cell failure in diabetes. Nat. Commun. 10, 3312. 10.1038/s41467-019-11170-4 31346174 PMC6658524

[B36] Persson F. Linden M. Unoson C. Elf J. (2013). Extracting intracellular diffusive states and transition rates from single-molecule tracking data. Nat. Methods 10, 265–269. 10.1038/nmeth.2367 23396281

[B37] Pierobon P. Achouri S. Courty S. Dunn A. R. Spudich J. A. Dahan M. (2009). Velocity, processivity, and individual steps of single myosin V molecules in live cells. Biophys. J. 96, 4268–4275. 10.1016/j.bpj.2009.02.045 19450497 PMC2712235

[B38] Pinaud F. Michalet X. Iyer G. Margeat E. Moore H. P. Weiss S. (2009). Dynamic partitioning of a glycosyl-phosphatidylinositol-anchored protein in glycosphingolipid-rich microdomains imaged by single-quantum dot tracking. Traffic 10, 691–712. 10.1111/j.1600-0854.2009.00902.x 19416475 PMC2766537

[B39] Pouli A. E. Emmanouilidou E. Zhao C. Wasmeier C. Hutton J. C. Rutter G. A. (1998). Secretory-granule dynamics visualized *in vivo* with a phogrin-green fluorescent protein chimaera. Biochem. J. 333 (1), 193–199. 10.1042/bj3330193 9639579 PMC1219572

[B40] Rorsman P. Rentröm E. (2003). Insulin granule dynamics in pancreatic beta cells. Diabetologia 46, 1029–1045. 10.1007/s00125-003-1153-1 12879249

[B41] Schindelin J. Arganda-Carreras I. Frise E. Kaynig V. Longair M. Pietzsch T. (2012). Fiji: an open-source platform for biological-image analysis. Nat. Methods 9, 676–682. 10.1038/nmeth.2019 22743772 PMC3855844

[B42] Seino S. Shibasaki T. Minami K. (2011). Dynamics of insulin secretion and the clinical implications for obesity and diabetes. J. Clin. Invest. 121, 2118–2125. 10.1172/JCI45680 21633180 PMC3104758

[B43] Shen H. Tauzin L. J. Baiyasi R. Wang W. Moringo N. Shuang B. (2017). Single particle tracking: from theory to biophysical applications. Chem. Rev. 117, 7331–7376. 10.1021/acs.chemrev.6b00815 28520419

[B44] Shin W. Ge L. Arpino G. Villarreal S. A. Hamid E. Liu H. (2018). Visualization of membrane pore in live cells reveals a dynamic-pore theory governing fusion and endocytosis. Cell 173, 934–945. 10.1016/j.cell.2018.02.062 29606354 PMC5935532

[B45] Sprague B. L. Pego R. L. Stavreva D. A. McNally J. G. (2004). Analysis of binding reactions by fluorescence recovery after photobleaching. Biophys. J. 86, 3473–3495. 10.1529/biophysj.103.026765 15189848 PMC1304253

[B46] Tabei S. M. Burov S. Kim H. Y. Kuznetsov A. Huynh T. Jureller J. (2013). Intracellular transport of insulin granules is a subordinated random walk. Proc. Natl. Acad. Sci. U A 110, 4911–4916. 10.1073/pnas.1221962110 PMC3612641 23479621

[B47] Takahashi N. Hatakeyama H. Okado H. Miwa A. Kishimoto T. Kojima T. (2004). Sequential exocytosis of insulin granules is associated with redistribution of SNAP25. J. Cell Biol. 165, 255–262. 10.1083/jcb.200312033 15117968 PMC2172050

[B48] Takahashi N. Sawada W. Noguchi J. Watanabe S. Ucar H. Hayashi-Takagi A. (2015). Two-photon fluorescence lifetime imaging of primed SNARE complexes in presynaptic terminals and beta cells. Nat. Commun. 6, 8531. 10.1038/ncomms9531 26439845 PMC4600761

[B49] Tarantino N. Tinevez J. Y. Crowell E. F. Boisson B. Henriques R. Mhlanga M. (2014). TNF and IL-1 exhibit distinct ubiquitin requirements for inducing NEMO-IKK supramolecular structures. J. Cell Biol. 204, 231–245. 10.1083/jcb.201307172 24446482 PMC3897181

[B50] Thurmond D. C. Gonelle-Gispert C. Furukawa M. Halban P. A. Pessin J. E. (2003). Glucose-stimulated insulin secretion is coupled to the interaction of actin with the t-SNARE (target membrane soluble N-ethylmaleimide-sensitive factor attachment protein receptor protein) complex. Mol. Endocrinol. 17, 732–742. 10.1210/me.2002-0333 12554769

[B51] Tinevez J. Y. Perry N. Schindelin J. Hoopes G. M. Reynolds G. D. Laplantine E. (2017). TrackMate: an open and extensible platform for single-particle tracking. Methods 115, 80–90. 10.1016/j.ymeth.2016.09.016 27713081

[B52] Varadi A. Tsuboi T. Johnson-Cadwell L. I. Allan V. J. Rutter G. A. (2003). Kinesin I and cytoplasmic dynein orchestrate glucose-stimulated insulin-containing vesicle movements in clonal MIN6 beta-cells. Biochem. Biophys. Res. Commun. 311, 272–282. 10.1016/j.bbrc.2003.09.208 14592410

[B53] Varadi A. Tsuboi T. Rutter G. A. (2005). Myosin Va transports dense core secretory vesicles in pancreatic MIN6 beta-cells. Mol. Biol. Cell 16, 2670–2680. 10.1091/mbc.e04-11-1001 15788565 PMC1142415

[B54] Wasmeier C. Hutton J. C. (1996). Molecular cloning of phogrin, a protein-tyrosine phosphatase homologue localized to insulin secretory granule membranes. J. Biol. Chem. 271, 18161–18170. 10.1074/jbc.271.30.18161 8663434

[B55] Xu S. Nam S. M. Kim J.-H. Das R. Choi S.-K. Nguyen T. T. (2015). Palmitate induces ER calcium depletion and apoptosis in mouse podocytes subsequent to mitochondrial oxidative stress. Cell Death Dis. 6, e1976. 10.1038/cddis.2015.331 26583319 PMC4670935

[B56] Zajac A. L. Goldman Y. E. Holzbaur E. L. F. Ostap E. M. (2013). Local cytoskeletal and organelle interactions impact molecular-motor-driven early endosomal trafficking. Curr. Biol. 23, 1173–1180. 10.1016/j.cub.2013.05.015 23770188 PMC3738301

