# Supplementary material for: Single-molecule analysis of intracellular insulin granule behavior and its application to analyzing cytoskeletal dependence and pathophysiological implications
Source: Front Physiol. 2023 Dec 6;14:1287275. doi: 10.3389/fphys.2023.1287275 (PMC10731264; doi:10.3389/fphys.2023.1287275)
Supplement: Supplementary file 2 [file DataSheet1.docx]

Supplementary Material

Single-molecule analysis of intracellular insulin granule behavior and its application to analyzing cytoskeletal dependence and pathophysiological implications

Hiroyasu Hatakeyama*, Tomomi Oshima, Shinichiro Ono, Yuichi Morimoto, Noriko Takahashi*

*** Correspondence:** Hiroyasu Hatakeyama: hatake@med.kitasato-u.ac.jp, Noriko Takahashi: ntakahas@med.kitasato-u.ac.jp

# Supplementary Figures


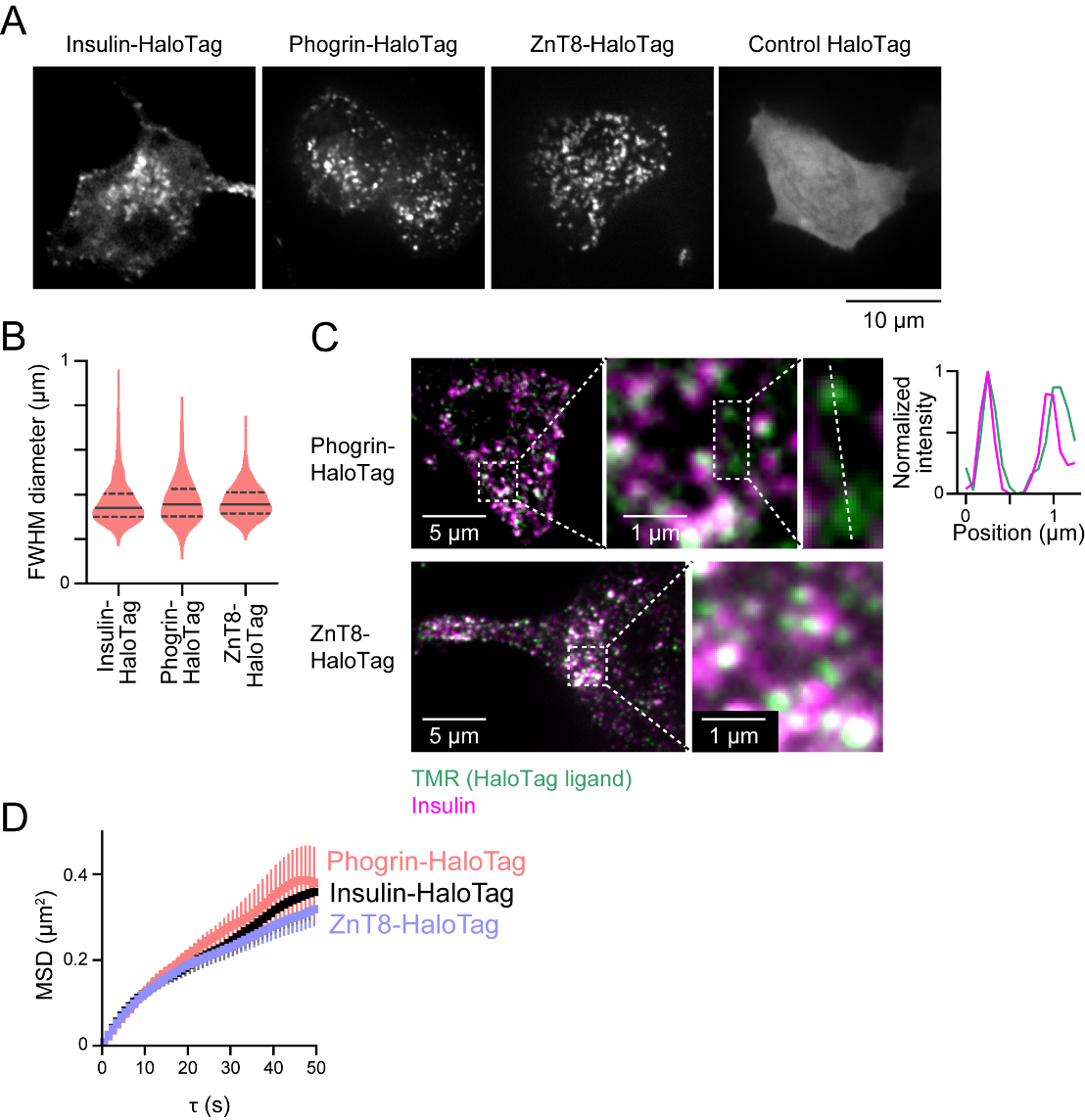


**Supplementary Figure 1.** Localization and size of HaloTag fusion proteins. (A) Snapshot of TMR fluorescence in INS-1 cells expressing the insulin-HaloTag, phogrin-HaloTag, ZnT8-HaloTag, and control HaloTag and stained with the HaloTag TMR ligand. (B) Violin plot of FWHM diameters of granular structures. Solid and dashed lines represent median and quartiles (upper and lower), respectively (n = 394–620 particles). The mean (± SEM) diameters were: insulin HaloTag; 0.365 ± 0.004 μm, phogrin-HaloTag; 0.370 ± 0.004 μm, and ZnT8-HaloTag; 0.367 ± 0.004 μm. (C) Immunofluorescence of insulin (magenta) in INS-1 cells expressing phogrin-HaloTag (left) or ZnT8-HaloTag (right) and staining with the HaloTag TMR ligand (green). Magnified images of boxed regions and intensity profiles along the dashed line are also shown. The mean (± SD) Manders’ colocalization coefficients M1 (fraction of overlapped TMR signals per total TMR signals) were: phogrin-HaloTag; 0.73 ± 0.17 (n = 17 cells), and ZnT8-HaloTag; 0.79 ± 0.09 (n = 16 cells). (D) Comparison of the intracellular behavior of insulin-HaloTag (black), phogrin-HaloTag (red), and ZnT8-HaloTag (blue) in INS-1 cells expressing the indicated proteins and stained with the HaloTag TMR ligand. Error bars represent SEM (n = 5–7 cells).


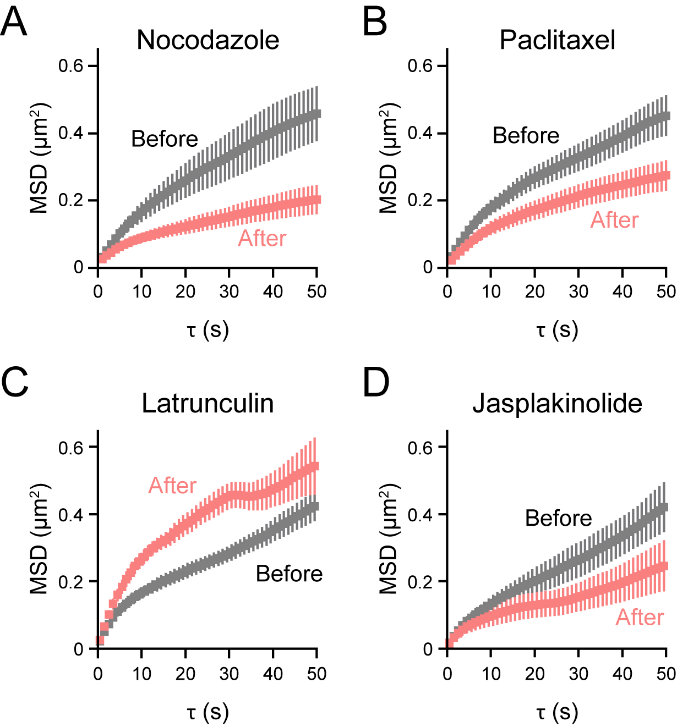
**Supplementary Figure 2.** Effect of microtubule and F-actin inhibitors on insulin granule movement. Mean MSD curves of insulin-HaloTag in INS-1 cells expressing the protein that had been stained with the HaloTag TMR ligand before (gray) and after (red) treatment with 3 μM nocodazole (A), 5 μM paclitaxel (B), 10 μM latrunculin B (C), and 1 μM jasplakinolide (D). Error bars represent SEM (n = 6–10 cells).
